# Ubiquinol decreases monocytic expression and DNA methylation of the pro-inflammatory chemokine ligand 2 gene in humans

**DOI:** 10.1186/1756-0500-5-540

**Published:** 2012-10-01

**Authors:** Alexandra Fischer, Simone Onur, Constance Schmelzer, Frank Döring

**Affiliations:** 1 Institute for Human Nutrition and Food Science, Department of Molecular Prevention, Christian Albrechts University, Kiel, Germany; 2 Research Unit Nutritional Physiology “Oskar Kellner”, Leibniz Institute for Farm Animal Biology (FBN), Dummerstorf, Germany

**Keywords:** Coenzyme Q10, Ubiquinol, Gene expression, DNA methylation, Inflammation

## Abstract

**Background:**

Coenzyme Q_10_ is an essential cofactor in the respiratory chain and serves in its reduced form, ubiquinol, as a potent antioxidant. Studies *in vitro* and *in vivo* provide evidence that ubiquinol reduces inflammatory processes via gene expression. Here we investigate the putative link between expression and DNA methylation of ubiquinol sensitive genes in monocytes obtained from human volunteers supplemented with 150 mg/ day ubiquinol for 14 days.

**Findings:**

Ubiquinol decreases the expression of the pro-inflammatory chemokine (C-X-C motif) ligand 2 gene (CXCL2) more than 10-fold. Bisulfite-/ MALDI-TOF-based analysis of regulatory regions of the CXCL2 gene identified six adjacent CpG islands which showed a 3.4-fold decrease of methylation status after ubiquinol supplementation. This effect seems to be rather gene specific, because ubiquinol reduced the expression of two other pro-inflammatory genes (PMAIP1, MMD) without changing the methylation pattern of the respective gene.

**Conclusion:**

In conclusion, ubiquinol decreases monocytic expression and DNA methylation of the pro-inflammatory CXCL2 gene in humans. Current Controlled Trials ISRCTN26780329.

## Background

Coenzyme Q_10_ (CoQ_10_) is a key component of the mitochondrial respiratory chain where it is mainly known for its role in oxidative phosphorylation. The reduced form of CoQ_10_, ubiquinol, serves as a potent antioxidant in mitochondria, lipid membranes and plasma lipoproteins [1,2] as well as a regenerator of other lipid soluble antioxidants (e.g. vitamin E) [3,4]. Several studies *in vitro*[5-7], in SAMP1 mice [8] and in humans [9] indicate that ubiquinol is involved in inflammatory processes and lipid metabolism via gene expression.

Gene expression as well as DNA methylation processes are affected by various dietary supplements and food nutrients [10-13]. Furthermore, DNA methylation is one of the epigenetic modifications that *per se* is able to determine the gene expression by regulating the chromatin organization [10,14]. During methylation of mammalian DNA a methyl group is attached at the 5-position of the cytosine residue within the cytosine-guanine dinucleotides (CpG) resulting in the formation of 5-methylcytosine, which is designated as the fifth base of DNA [15]. Although most genomic DNA in mammals is deficient in CpG sites, clusters of CpG dinucleotides (CpG islands) were described to be primarily located at promoter regions of genes [16]. Here we investigated the effect of ubiquinol on the expression and methylation of CpG island promoter regions of pro-inflammatory genes in humans.

### Materials and methods

#### Participants and study design

Sample characteristics of subjects and study design have been described lately [9]. Briefly: fifty-three healthy male volunteers, 21–48 years of age, received 150 mg of the reduced form of CoQ10 (Q_10_H_2_, ubiquinol, KANEKA Corporation, Japan) daily in form of three capsules with each principal meal for 14 days. Fasting blood samples were taken before (T_0_) and after (T_14_) supplementation. The participants had an average Body Mass Index (BMI) of 24.1 ± 2.5 kg/m^2^, no history of gastrointestinal, hepatic, cardiovascular or renal diseases, a habit of non- or occasional smoking and maintenance of usual nutrition habits. The study was approved by the ethics committee of the Medical Faculty of Kiel University, Germany, and was conformed to Helsinki Declaration. All volunteers gave written informed consent.

#### Microarray-based gene expression analysis and qRT-PCR

Microarray experiment using the Affymetrix human genome U133 plus 2.0 GeneChip® were performed as described previously [5] with RNA samples from CD14-positive monocytes obtained from three volunteers before (T_0_) and after (T_14_) supplementation with ubiquinol. Based on microarray data, expression levels of selected genes including the CXCL2, MMD and PMAIP1 gene were verified by real-time qRT-PCR. Primer sequences for real-time qRT-PCR experiments were designed with Primer Express® Software 3.0 (Applied Biosystems, Darmstadt, Germany). Primer pairs were obtained from MWG Biotech AG (Ebersberg, Germany). cDNA synthesis with subsequent PCR amplification procedure has been described before [9].

#### Methylation analysis of genomic regions of CXCL2, MMD and PMAIP1 gene

The presence of CpG islands within the CXCL2, MMD and PMAIP1 genes was predicted using the European Molecular Biology Open Software Suite CpGplot, respectively. Quantitative methylation analysis was performed on the MassARRAY® system (Sequenom, Hamburg, Germany) at BioGlobe (Hamburg, Germany) applying the MassCLEAVE^TM^ (hMC) biochemistry after bisulfit treatment of DNA samples and MALDI-TOF mass spectrometry for analyte detection. All reactions were performed according to the standard protocols recommended by the supplier. Genomic DNA was extracted from human monocytes obtained from five volunteers (H1/H1_1 to H5/H5_1) before (T_0_, H1-H5) and after (T_14_, H1_1-H5_5) using the DNeasy Tissue Kit (Quiagen). Analysis was carried out from both, forward and reverse strand.

The protocol starts with a bisulfit treatment of provided genomic DNA sample, which converts native cytosine (“C”) nucleotides into uracil (“U”), whereas 5-methyl-protected cytosine residues remain as “C”. The resulting artificial sequence variation is conserved during PCR amplification using methylation independent primers. One primer for each PCR is tagged with T7 RNA polymerase promoter sequence facilitating the transformation of double stranded PCR product into single stranded RNA together with a second level of amplification. The *in vitro* transcription product is “U-specific” cleaved with RNase A. The generated fragments represent unique portions of the amplified region of interest and are displayed based on their molecular weight in the mass spectrum, which is acquired after sample conditioning with a MassARRAY® Analyzer Compact. Automated data analysis was performed with EpiTyper Software.

## Findings

### Results and discussion

#### Ubiquinol supplementation reduces the expression of low and high abundant mRNA steady-state levels of pro-inflammatory genes in human monocytes

Several studies *in vitro* and in rodents indicate that Coenzyme Q_10_ or rather its reduced form, ubiquinol, reduces inflammatory processes via gene expression. To study the putative link between ubiquinol dependent gene expression and DNA methylation we used probes from our human study [9]. In this study, we found a significant decrease of LDL-cholesterol and erythropoiesis after a 14 day supplementation period with 150 mg/ day ubiquinol. Microarray-based gene expression analysis and qRT-PCR verification of selected genes identified 272 genes regulated by ubiquinol supplementation in monocytes. This gene list was used to select pro-inflammatory genes which differ in their expression levels at baseline (T_0_) and showed markedly differences in ubiquinol dependent regulation. Therefore, three genes encoding the chemokine (C-X-C motif) ligand 2 (CXCL2), the phorbol-12-myristate-13-acetate-induced protein 1 (PMAIP1) and the monocyte to macrophage differentiation-associated protein (MMD) were selected. CXCL2 as well as PMAIP1 and MMD are involved in differentiation processes of blood monocytes to macrophages [17-20]. Furthermore, CXCL2 and PMAIP1 are key players in apoptosis induction and inflammatory responses, respectively [21-23]. The MMD gene is highly expressed in mature macrophages but its exact biological function is not clear so far [24]. As shown in Table 1, PMAIP1 and CXCL2 showed about 8.7 to 7.9-fold higher expression levels than MMD gene at baseline (T_0_) in human monocytes. Ubiquinol supplementation leads to a down regulation of the low expressing MMD gene by a factor of 1.7. The expression of the high abundant transcript of the PMAIP gene is 2.2-fold reduced by ubiquinol. Remarkably, ubiquinol decreases the expression of the high expressing CXCL2 gene more than 10-fold. Together, ubiquinol reduces the expression of the pro-inflammatory genes CXCL2, PMAIP1 and MMD.

**Table 1:** **Normalized steady-state mRNA expression levels (AU) of the CXCL2, PMAIP1 and MMD gene in monocytes of human volunteers before (T**_**0**_**) and after (T**_**14**_**) supplementation with ubiquinol**

**Gene**	**T0**	**T14**	**Fold change**
			**(T0 vs. T14)**
**PMAIP1**	1271 ± 35	578 ± 106	-2,2
**MMD**	147 ± 30	85 ± 19	-1,7
**CXCL2**	1150 ± 865	93 ± 30	-12,4

T_0_, expression levels before supplementation (baseline); T_14_, expression levels after a 14 day supplementation period with 150 mg/ d ubiquinol; fold change, relative change from time point T_0_ to T_14_; AU, arbitrary units; Data are given as mean ± SEM.

#### Ubiquinol supplementation reduces the methylation status of six adjacent CpG islands within the promoter of the CXCL2 gene

**Figure 1:**
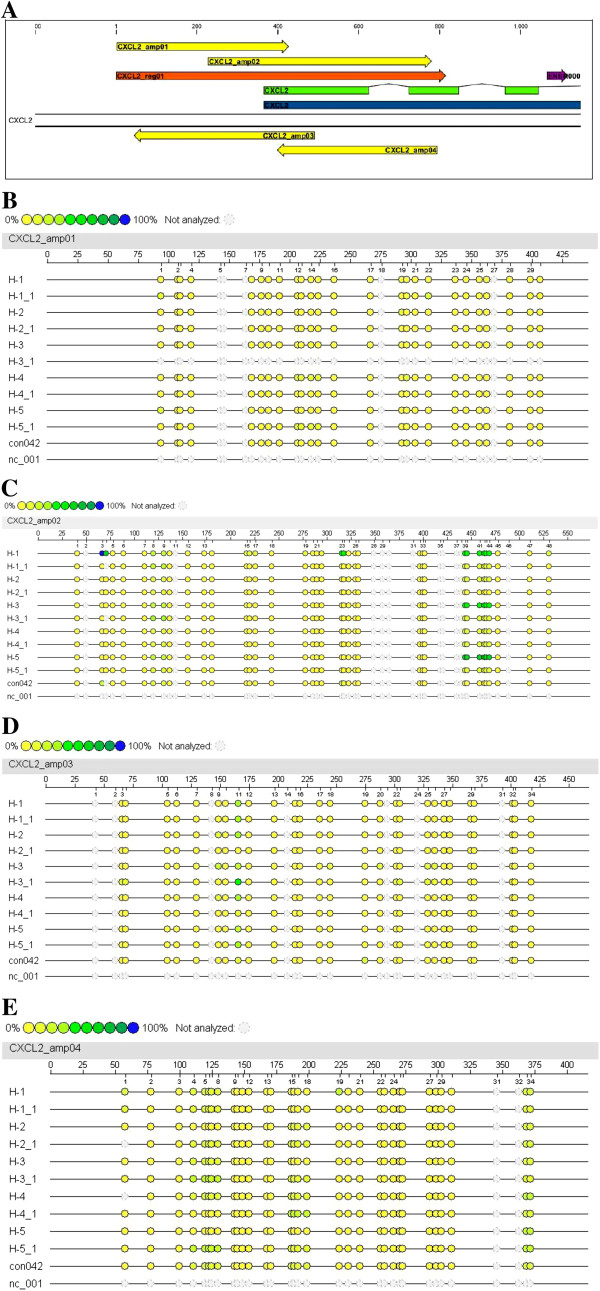
**Position of amplicons within the analysed genomic region (A) and methylation status of CpG islands (B-E) of the human CXCL2 gene. A**, The genomic region of the CXCL2 gene is located from −365 to +450 relative to the gene start. This refers to position 74964548–74965362 of the NCBI’s human genome build 37.1. The gene is shown in its transcripted orientation and locates on the sense strand of chromosome 4. Colors illustrate position of the gene (blue), mRNA (green), region for amplicon design (orange), amplicons (yellow) and annotated (Ensembl) regulatory region (pink). **B**-**E**, Colored dots indicate the methylation ratio (%) at each analyzed CpG-unit within each amplicon. Samples are indicated as H-1 to H-5 (time point T_0_) and H-1_1 to H-5_1 (T_14_). Base count (upper ruler scale) and CpG-site numbering (lower ruler scale) refers to the analyzed strand in 5’→3’ orientation of the analyzed amplicon sequence. Sample “nc_001” represents the reaction negative control (water) and “con42” a control DNA.

**Figure 2:**
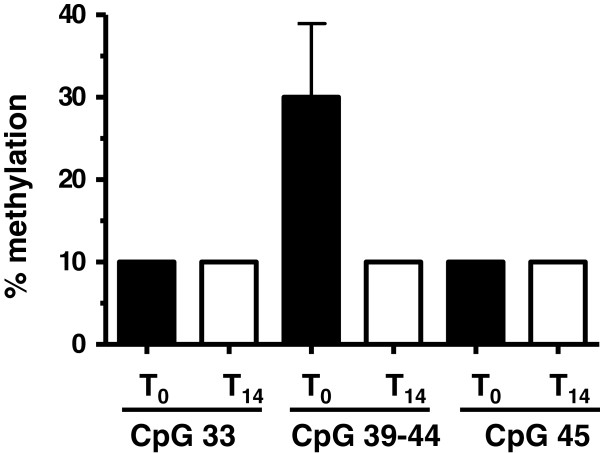
**Methylation status of CpG islands of the CXCL2 gene in monocytes of human volunteers before (T**_**0**_**) and after (T**_**14**_**) a 14 day supplementation period with ubiquinol. **The extent of methylation (%, mean ± SD) of CpG islands 33, 39–44 and 45 within amplicon 2 of the CXCL2 gene (see Figure 1C) is shown.

There is evidence that difference in gene expression correlates with CpG island variation [25]. In order to evaluate whether the ubiquinol dependent reduction in the expression of the CXCL2, PMAIP1 and MMD gene are linked to variation in methylation patterns, bisulfit-based and matrix-assisted laser desorption ionization time-of-flight (MALDI-TOF) mass spectrometry was used for analysing CpG islands within promoter regions of the respective genes. As shown in Table 2 and Figure 1A/ Additional file 1: Figure S1A/ Additional file 2: Figure S2A, we analysed in total 656 CpG islands which covered the promoter regions and the adjacent exon 1 of the CXCL2 (146 CpG islands), PMAIP1 (347) and MMD (163) gene. There was only weak methylation detected in the analysed CpG islands of the genes PMAIP1 and MMD before and after supplementation with ubiquinol (Additional file 1: Figure S1B-H, Additional file 2: Figure S2B/C). Most CpG islands of the CXCL2 gene were also unaffected by ubiquinol (Figure 1B/D/E). As shown in Figure 1C, the methylation status of six adjacent CpG islands (39 to 44) was reduced under ubiquinol treatment. Methylation pattern of CpG islands which are located in close proximity to this region showed no alteration before and after ubiquinol supplementation. Quantitative analysis revealed that ubiquinol reduced the methylation of CpG islands 39–44 (Figure 2) of the CXCL2 gene by a factor of 3.4.

**Table 2:** Position, length and number of CpG islands of amplicons covering the analysed genomic regions of the human CXCL2, PMAIP1 and MMD gene

	**start**	**end**	**length**	**CpGs**	**left primer (+Tag)**	**right primer (+Tag)**
***gene/amplicon***						
**PMAIP1_amp01 *f***	3	565	563	57	ATTGTTAAGGTTTTTGGTTTTTTTT	CTCAACCTCCAACTAAAACACCTC
**PMAIP1_amp02 *f***	485	1068	584	49	TTTTAGTATTTTTGTTTGTAGGATTGTT	AAACTCTCTCCTACCCCTTCTACC
**PMAIP1_amp03 *f***	1113	1529	417	13	AGGGTTTTTGTGTTTAGGAGTTTAGA	AAATAAACAAAACTTTTTCCATCCC
**PMAIP1_amp07 *f***	-23	565	587	57	TTTGGGTTTGTTTATTTAAGTTTTT	CTCAACCTCCAACTAAAACACCTC
**PMAIP1_amp04 *r***	3	512	510	53	AATAGTTTTGTAGGTAGGGATGTTGG	ATTACCAAAACCTCTAATCTCTCCC
**PMAIP1_amp05 *r***	374	954	581	53	AGGAGGAAAGGAGTTTTTTGTTTTT	TCACCAAAAAAATTCTCACTAAACA
**PMAIP1_amp06 *r***	930	1481	552	22	TTGTTATTAATTTAGGTATGGTTATATTTG	AAAAACAAAAAACTCCTTTCCTCCT
**PMAIP1_amp08 *r***	-9	399	409	43	TTGTTTAGTGAGAATTTTTTTGGTG	CCCAAATCTCTAATTACCAAAACCT
**MMD_amp01 *f***	236	986	751	81	AGGTAGGGTTGTTTGTTTGTTGTTA	AATCCACCCAAAATAAATCCAAAT
**MMD_amp02 *r***	274	1015	742	82	TAGGGAATTGATTTTTGGTTAAGGT	ACTTTTAAAATTTCCTAATCCATCTCC
**CXCL2_amp01 *f***	2	427	426	30	AGGATSGSTAAGATATGTTGTAGTTTTTG	CTTTTATACATAATTAAAACTAAAAAACCC
**CXCL2_amp02 *f***	228	780	553	48	GGGGTAGAAAGAGAATATTTTATAGTTGG	AAATTCCCTACAAAATCTACAAACAC
**CXCL2_amp03 *r***	45	491	447	34	GAGGAGAGTTGGTAAGGAGTTGTTT	CCCAACAACTAAAATATCTTCCAAAA
**CXCL2_amp04 *r***	399	794	396	34	GATGTTTTTGAGGTGAATTTTTTGT	AACTTTCCAACCCCAACCATACATA

The start and end positions of amplicons refer to the genomic regions as illustrated in Figure 1 (CXCL2 gene) and Additional file 1: Figure S1 (PMAIP1) and Additional file 2: Figure S2 (MMD). Primer sequences shown in 5´ to 3´ direction are complementary to sequences obtained after bisulfite treatment and differ from the original genomic target by exchanging each “C” with “T”. f, forward direction of the amplicon; r, reverse direction of the amplicon.

#### The effect of ubiquinol on DNA methylation seems to be rather gene specific and might depend on the extent of ubiquinol induced alteration of gene expression

Our study provides a first hint towards a modifying effect of ubiquinol on DNA methylation in humans. This effect is in line with another human study demonstrating that a supplementation with a mixture of CoQ_10_, niacin and riboflavin reduces DNA methylation of the tumor suppressor gene RASSF1A in breast cancer patients undergoing tamoxifen therapy [26]. Moreover, literature indicates that global methylation patterns are affected by several other dietary supplements and micronutrients [27-32]. The effect of ubiquinol on DNA methylation seems be rather gene specific because we identified two genes (PMAIP1, MMD) which are regulated by ubiquinol without changing DNA methylation. A recent SAMP-mice study from our group [33] found that ubiquinol alters hepatic expression of PPARα target genes without influencing DNA methylation in the respective gene promoters.

The effect of ubiquinol on DNA methylation might be linked to the extent of ubiquinol dependent alteration of gene expression. In the case of the CXCL2 gene, ubiquinol reduces its expression more than 10-fold accompanied by a reduced methylation status within certain CpG islands. This finding seems to be contradictory to common models of gene expression, because increased DNA methylation of a gene leads to reduced expression levels [34], whereas demethylation correlates with the transcription of the gene [35]. On the other hand there is evidence, especially in cancer cells, that DNA methylation status does not correlate with gene expression [36,37]. However, the mechanism regarding effects of ubiquinol on DNA methylation and expression remains unclear and has to be studied in the future. The reduced expression of pro-inflammatory genes under ubiquinol supplementation supports recent findings from our lab [5,6,8,9,38-41] and other groups [42,43] suggesting that CoQ_10_ displays anti-inflammatory properties. As a summary, we found in a human intervention study that ubiquinol decreases expression and DNA methylation of the pro-inflammatory CXCL2 gene in monocytes. Further studies will be necessary to investigate the mechanistic link between ubiquinol dependent gene expression, DNA methylation and inflammation.

## Competing interest

All authors declare that they have no competing interests.

## Authors’ contribution

AF and CS carried out the experiments and collected the data. SO analysed the data and wrote the manuscript. FD designed the study and drafted the manuscript. All authors read and approved the final manuscript.

## Supplementary Material

Additional file 1 **Figure S1. **Position of amplicons within the analysed genomic region (A) and methylation status of CpG islands (B-H) of the human PMAIP gene. A, The genomic region of the PMAIP gene is located from −390 to +1153 relative to the gene start. This refers to position 57566802–57568344 of the NCBI’s human genome build 37.1. The gene is shown in its transcripted orientation and locates on the sense strand of chromosome 18. Colors illustrate position of the gene (blue), mRNA (green), region for amplicon design (orange), amplicons (yellow) and annotated (Ensembl) regulatory region (pink). B-H, Colored dots indicate the methylation ratio (%) at each analyzed CpG-unit within each amplicon. Samples are indicated as H-1 to H-5 (time point T_0_) and H-1_1 to H-5_1 (T_14_). Base count (upper ruler scale) and CpG-site numbering (lower ruler scale) refers to the analyzed strand in 5’→3’ orientation of the analyzed amplicon sequence. Sample “nc_001” represents the reaction negative control (water) and “con42” a control DNA. Click here for file

Additional file 2 **Figure S2.** Position of amplicons with in the analysed genomic region (A) and methylation status of CpG islands (B, C) of the human MMD gene. A, The genomic region of the MMD gene is located from −564 to +470 relative to the gene start. This refers to position 53498872–53499905 of the NCBI’s human genome build 37.1. The gene is shown in its transcripted orientation and locates on the sense strand of chromosome 17. Colors illustrate position of the gene (blue), mRNA (green), region for amplicon design (orange), amplicons (yellow) and annotated (Ensembl) regulatory region (pink). B and C, Colored dots indicate the methylation ratio (%) at each analyzed CpG-unit within each amplicon. Samples are indicated as H-1 to H-5 (time point T_0_) and H-1_1 to H-5_1 (T_14_). Base count (upper ruler scale) and CpG-site numbering (lower ruler scale) refers to the analyzed strand in 5’→3’ orientation of the analyzed amplicon sequence. Sample “nc_001” represents the reaction negative control (water) and “con42” a control DNA. Click here for file

## References

[B1] Littarru GP Tiano L Bioenergetic and antioxidant properties of coenzyme Q10: recent developments Mol Biotechnol 2007 37 1 31 37 10.1007/s12033-007-0052-y 17914161

[B2] Littarru GP Tiano L Clinical aspects of coenzyme Q10: an update Nutrition 2010 26 3 250 254 10.1016/j.nut.2009.08.008 19932599

[B3] Mukai K Itoh S Morimoto H Stopped-flow kinetic study of vitamin E regeneration reaction with biological hydroquinones (reduced forms of ubiquinone, vitamin K, and tocopherolquinone) in solution J Biol Chem 1992 267 31 22277 22281 1429580

[B4] Crane FL Navas P The diversity of coenzyme Q function Mol Aspects Med 1997 18 Suppl S1 S6 9266500 10.1016/s0098-2997(97)00016-2

[B5] Schmelzer C Doring F Identification of LPS-inducible genes downregulated by ubiquinone in human THP-1 monocytes Biofactors 2010 36 3 222 228 10.1002/biof.93 20533395

[B6] Schmelzer C Kohl C Rimbach G Doring F The reduced form of coenzyme Q10 decreases the expression of lipopolysaccharide-sensitive genes in human THP-1 cells J Med Food 2011 14 4 391 397 10.1089/jmf.2010.0080 21370964

[B7] Groneberg DA Kindermann B Althammer M Klapper M Vormann J Littarru GP Doring F Coenzyme Q10 affects expression of genes involved in cell signalling, metabolism and transport in human CaCo-2 cells Int J Biochem Cell Biol 2005 37 6 1208 1218 10.1016/j.biocel.2004.11.017 15778085

[B8] Schmelzer C Kubo H Mori M Sawashita J Kitano M Hosoe K Boomgaarden I Doring F Higuchi K Supplementation with the reduced form of Coenzyme Q10 decelerates phenotypic characteristics of senescence and induces a peroxisome proliferator-activated receptor-alpha gene expression signature in SAMP1 mice Mol Nutr Food Res 2010 54 6 805 815 19960455 10.1002/mnfr.200900155

[B9] Schmelzer C Niklowitz P Okun JG Haas D Menke T Doring F Ubiquinol-induced gene expression signatures are translated into altered parameters of erythropoiesis and reduced low density lipoprotein cholesterol levels in humans IUBMB Life 2011 63 1 42 48 10.1002/iub.413 21280176

[B10] Vucetic Z Kimmel J Totoki K Hollenbeck E Reyes TM Maternal high-fat diet alters methylation and gene expression of dopamine and opioid-related genes Endocrinology 2010 151 10 4756 4764 10.1210/en.2010-0505 20685869 PMC2946145

[B11] Yubero-Serrano EM Gonzalez-Guardia L Rangel-Zuniga O Delgado-Lista J Gutierrez-Mariscal FM Perez-Martinez P Delgado-Casado N Cruz-Teno C Tinahones FJ Villalba JM Mediterranean diet supplemented with coenzyme Q10 modifies the expression of proinflammatory and endoplasmic reticulum stress-related genes in elderly men and women J Gerontol A: Biol Sci Med Sci 2012 67 1 3 10 22016358 10.1093/gerona/glr167

[B12] van den Donk M van Engeland M Pellis L Witteman BJ Kok FJ Keijer J Kampman E Dietary folate intake in combination with MTHFR C677T genotype and promoter methylation of tumor suppressor and DNA repair genes in sporadic colorectal adenomas Cancer epidemiology, biomarkers & prevention: a publication of the American Association for Cancer Research, cosponsored by the American Society of Preventive Oncology 2007 16 2 327 333 10.1158/1055-9965.EPI-06-0810 17301267

[B13] van Engeland M Weijenberg MP Roemen GM Brink M de Bruine AP Goldbohm RA van den Brandt PA Baylin SB de Goeij AF Herman JG Effects of dietary folate and alcohol intake on promoter methylation in sporadic colorectal cancer: the Netherlands cohort study on diet and cancer Cancer Res 2003 63 12 3133 3137 12810640

[B14] Silahtaroglu A Stenvang J MicroRNAs, epigenetics and disease Essays Biochem 2010 48 1 165 185 10.1042/bse0480165 20822493

[B15] Delaval K Feil R Epigenetic regulation of mammalian genomic imprinting Curr Opin Genet Dev 2004 14 2 188 195 10.1016/j.gde.2004.01.005 15196466

[B16] Takai D Jones PA Comprehensive analysis of CpG islands in human chromosomes 21 and 22 Proc Natl Acad Sci USA 2002 99 6 3740 3745 10.1073/pnas.052410099 11891299 PMC122594

[B17] Bender AT Beavo JA PDE1B2 regulates cGMP and a subset of the phenotypic characteristics acquired upon macrophage differentiation from a monocyte Proc Natl Acad Sci USA 2006 103 2 460 465 10.1073/pnas.0509972102 16407168 PMC1326187

[B18] Bourdonnay E Morzadec C Sparfel L Galibert MD Jouneau S Martin-Chouly C Fardel O Vernhet L Global effects of inorganic arsenic on gene expression profile in human macrophages Mol Immunol 2009 46 4 649 656 10.1016/j.molimm.2008.08.268 19128835

[B19] Liang F Seyrantepe V Landry K Ahmad R Ahmad A Stamatos NM Pshezhetsky AV Monocyte differentiation up-regulates the expression of the lysosomal sialidase, Neu1, and triggers its targeting to the plasma membrane via major histocompatibility complex class II-positive compartments J Biol Chem 2006 281 37 27526 27538 10.1074/jbc.M605633200 16835219

[B20] Liu Q Zheng J Yin DD Xiang J He F Wang YC Liang L Qin HY Liu L Liang YM Han H Monocyte to macrophage differentiation-associated (MMD) positively regulates ERK and Akt activation and TNF-alpha and NO production in macrophages Mol Biol Rep 2012 39 5 5643 5650 10.1007/s11033-011-1370-5 22203480

[B21] Yu J Zhang L The transcriptional targets of p53 in apoptosis control Biochem Biophys Res Commun 2005 331 3 851 858 10.1016/j.bbrc.2005.03.189 15865941

[B22] Kim HY Kim HS Upregulation of MIP-2 (CXCL2) expression by 15-deoxy-Delta(12,14)-prostaglandin J(2) in mouse peritoneal macrophages Immunol Cell Biol 2007 85 1 60 67 10.1038/sj.icb.7100001 17130903

[B23] Lkhagvaa B Tani K Sato K Toyoda Y Suzuka C Sone S Bestatin, an inhibitor for aminopeptidases, modulates the production of cytokines and chemokines by activated monocytes and macrophages Cytokine 2008 44 3 386 391 10.1016/j.cyto.2008.10.011 19036603

[B24] Rehli M Krause SW Schwarzfischer L Kreutz M Andreesen R Molecular cloning of a novel macrophage maturation-associated transcript encoding a protein with several potential transmembrane domains Biochem Biophys Res Commun 1995 217 2 661 667 10.1006/bbrc.1995.2825 7503749

[B25] Shen L Kondo Y Guo Y Zhang J Zhang L Ahmed S Shu J Chen X Waterland RA Issa JP Genome-wide profiling of DNA methylation reveals a class of normally methylated CpG island promoters PLoS Genet 2007 3 10 2023 2036 17967063 10.1371/journal.pgen.0030181 PMC2041996

[B26] Premkumar VG Yuvaraj S Shanthi P Sachdanandam P Co-enzyme Q10, riboflavin and niacin supplementation on alteration of DNA repair enzyme and DNA methylation in breast cancer patients undergoing tamoxifen therapy Br J Nutr 2008 100 6 1179 1182 10.1017/S0007114508968276 18377693

[B27] Brunaud L Alberto JM Ayav A Gerard P Namour F Antunes L Braun M Bronowicki JP Bresler L Gueant JL Effects of vitamin B12 and folate deficiencies on DNA methylation and carcinogenesis in rat liver Clin Chem Lab Med: CCLM / FESCC 2003 41 8 1012 1019 10.1515/CCLM.2003.155 12964806

[B28] Fischer A Gaedicke S Frank J Doring F Rimbach G Dietary vitamin E deficiency does not affect global and specific DNA methylation patterns in rat liver Br J Nutr 2010 104 7 935 940 10.1017/S0007114510001649 20447326

[B29] Ly A Hoyt L Crowell J Kim YI Folate and DNA Methylation Antioxid Redox Signal 2012 10.1089/ars.2012.4554 22332737

[B30] Uekawa A Katsushima K Ogata A Kawata T Maeda N Kobayashi K Maekawa A Tadokoro T Yamamoto Y Change of epigenetic control of cystathionine beta-synthase gene expression through dietary vitamin B12 is not recovered by methionine supplementation J Nutrigenet Nutrigenomics 2009 2 1 29 36 10.1159/000165374 19776636

[B31] Waterland RA Assessing the effects of high methionine intake on DNA methylation J Nutr 2006 136 6 Suppl 1706S 1710S 16702343 10.1093/jn/136.6.1706S

[B32] Wolff GL Kodell RL Moore SR Cooney CA Maternal epigenetics and methyl supplements affect agouti gene expression in Avy/a mice FASEB J: Off Publ Fed Am Soc Exp Biol 1998 12 11 949 957 9707167

[B33] Schmelzer C Okun JG Haas D Higuchi K Sawashita J Mori M Doring F The reduced form of coenzyme Q10 mediates distinct effects on cholesterol metabolism at the transcriptional and metabolite level in SAMP1 mice IUBMB Life 2010 62 11 812 818 10.1002/iub.388 21086475

[B34] Ehrlich M Expression of various genes is controlled by DNA methylation during mammalian development J Cell Biochem 2003 88 5 899 910 10.1002/jcb.10464 12616529

[B35] De Smet C Lurquin C Lethe B Martelange V Boon T DNA methylation is the primary silencing mechanism for a set of germ line- and tumor-specific genes with a CpG-rich promoter Mol Cell Biol 1999 19 11 7327 7335 10523621 10.1128/mcb.19.11.7327 PMC84726

[B36] Gama-Sosa MA Slagel VA Trewyn RW Oxenhandler R Kuo KC Gehrke CW Ehrlich M The 5-methylcytosine content of DNA from human tumors Nucleic Acids Res 1983 11 19 6883 6894 10.1093/nar/11.19.6883 6314264 PMC326421

[B37] Baylin SB Herman JG Graff JR Vertino PM Issa JP Alterations in DNA methylation: a fundamental aspect of neoplasia Adv Cancer Res 1998 72 141 196 9338076

[B38] Schmelzer C Kitano M Rimbach G Niklowitz P Menke T Hosoe K Doring F Effects of ubiquinol-10 on microRNA-146a expression in vitro and in vivo Mediators Inflamm 2009 2009 415437 19390647 10.1155/2009/415437 PMC2672161

[B39] Schmelzer C Lindner I Rimbach G Niklowitz P Menke T Doring F Functions of coenzyme Q10 in inflammation and gene expression Biofactors 2008 32 1–4 179 183 19096114 10.1002/biof.5520320121

[B40] Schmelzer C Lindner I Vock C Fujii K Doring F Functional connections and pathways of coenzyme Q10-inducible genes: an in-silico study IUBMB Life 2007 59 10 628 633 10.1080/15216540701545991 17852568

[B41] Schmelzer C Lorenz G Lindner I Rimbach G Niklowitz P Menke T Doring F Effects of Coenzyme Q10 on TNF-alpha secretion in human and murine monocytic cell lines Biofactors 2007 31 1 35 41 10.1002/biof.5520310104 18806307

[B42] Dominguez PM Ardavin C Differentiation and function of mouse monocyte-derived dendritic cells in steady state and inflammation Immunol Rev 2010 234 1 90 104 10.1111/j.0105-2896.2009.00876.x 20193014

[B43] Sohet FM Neyrinck AM Pachikian BD de Backer FC Bindels LB Niklowitz P Menke T Cani PD Delzenne NM Coenzyme Q10 supplementation lowers hepatic oxidative stress and inflammation associated with diet-induced obesity in mice Biochem Pharmacol 2009 78 11 1391 1400 10.1016/j.bcp.2009.07.008 19632207

