# Supplementary figures and images for: Ubiquinol decreases monocytic expression and DNA methylation of the pro-inflammatory chemokine ligand 2 gene in humans
Source: BMC Res Notes. 2012 Oct 1;5:540. doi: 10.1186/1756-0500-5-540 (PMC3542089; doi:10.1186/1756-0500-5-540)

**Figure S1 (PMAIP1)**

**A**

**
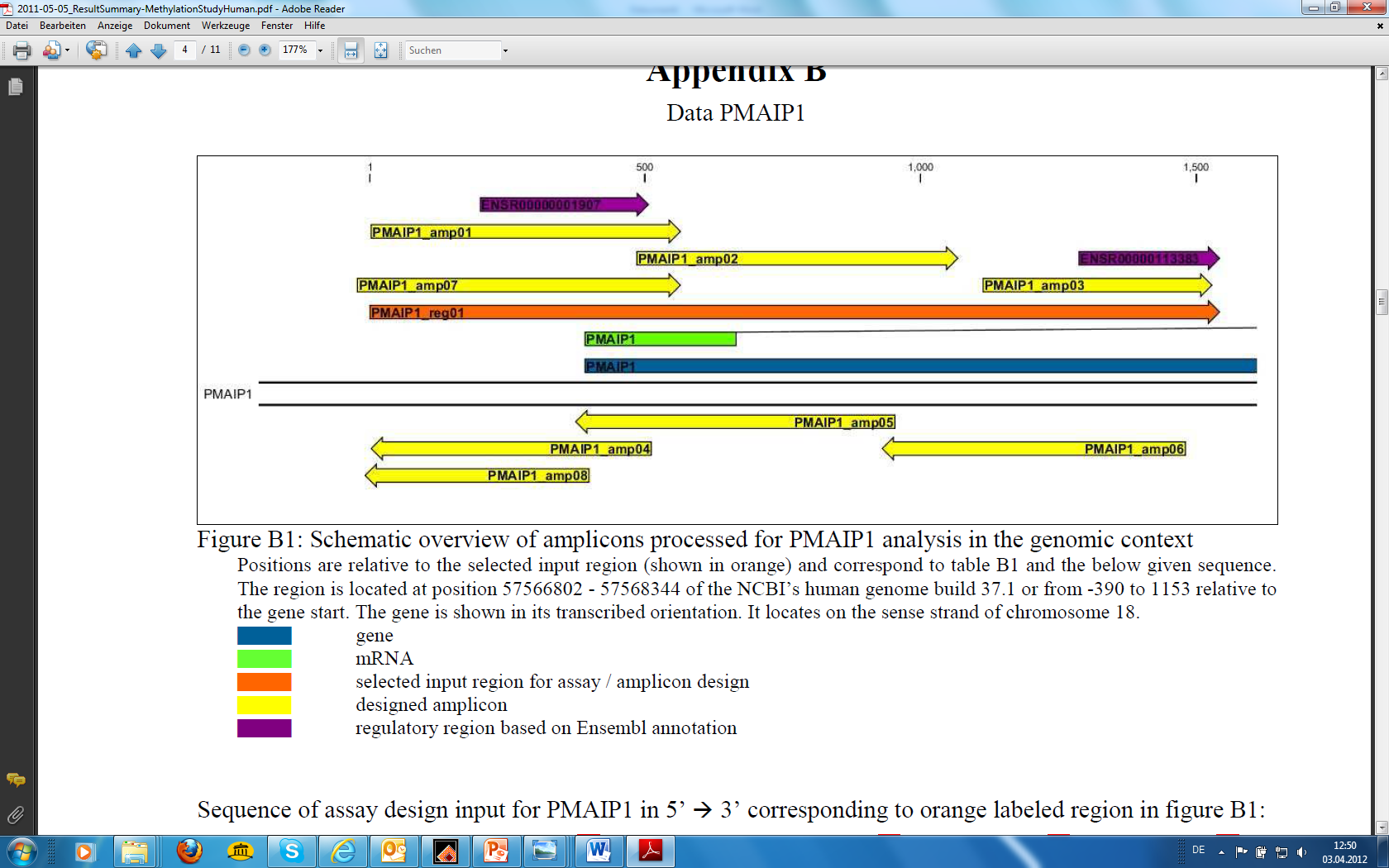
**

**B**

**
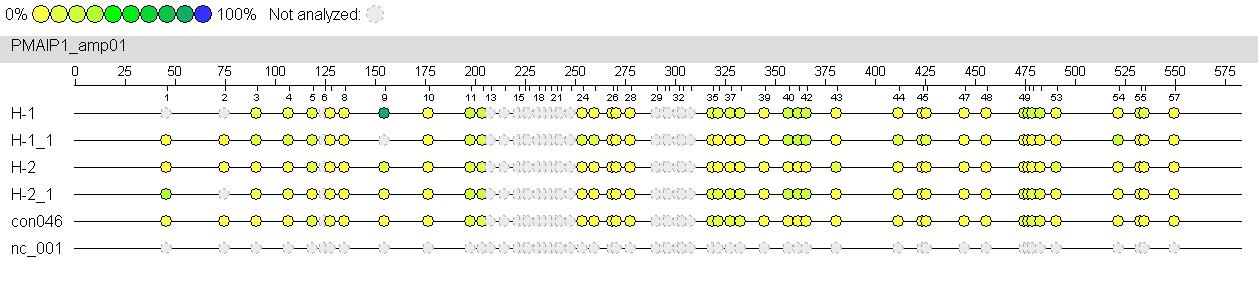
**

**C**

**
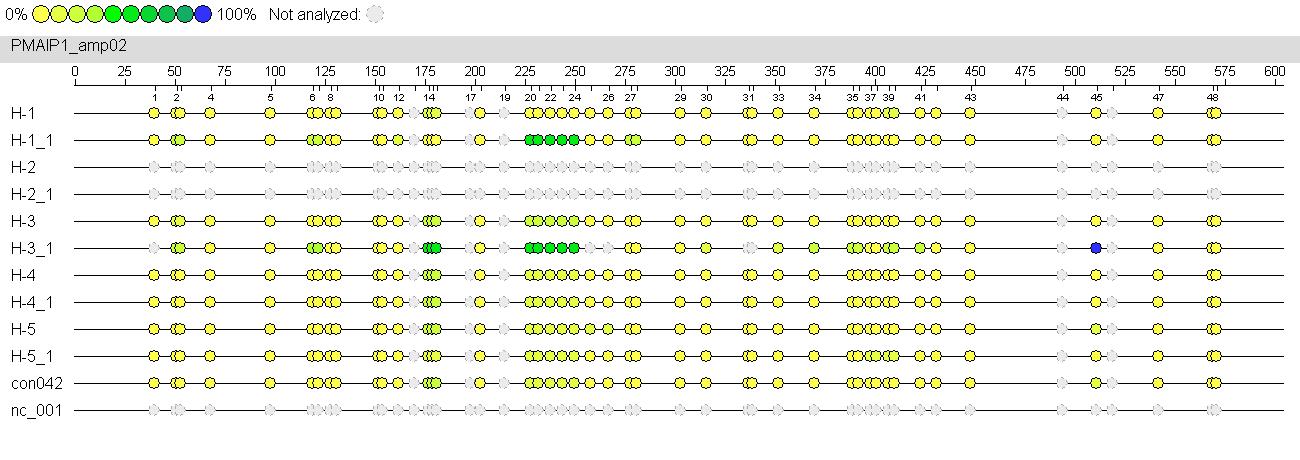
**

**D**

**
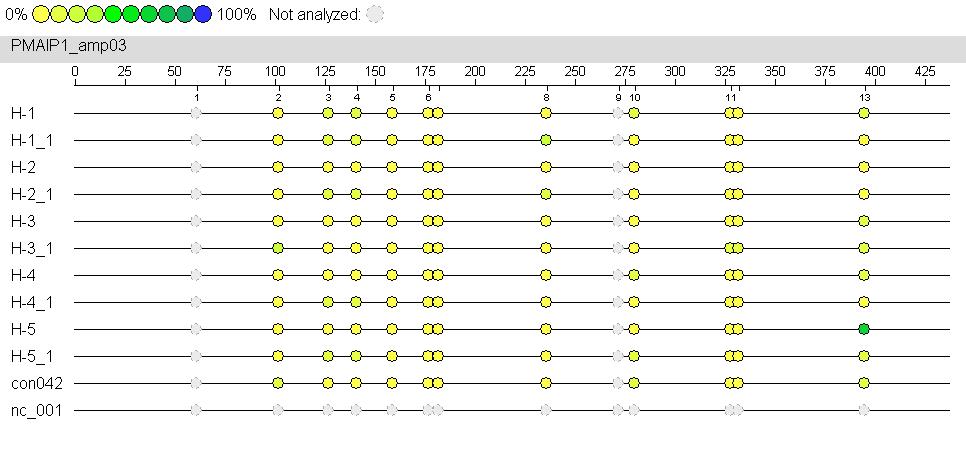
**

**E
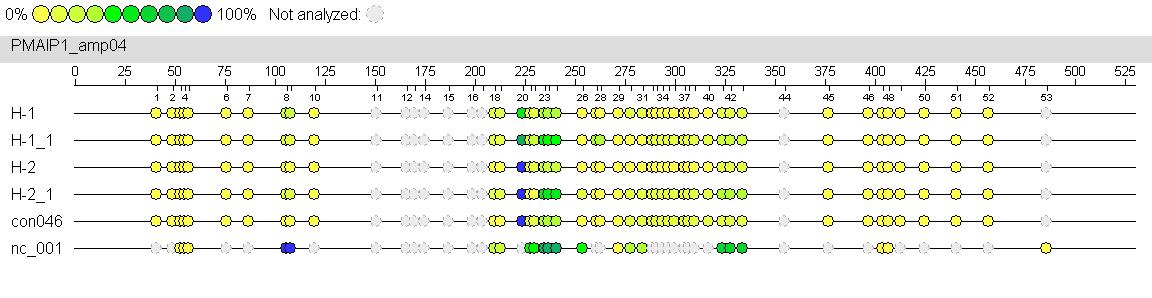
**

**F**

**
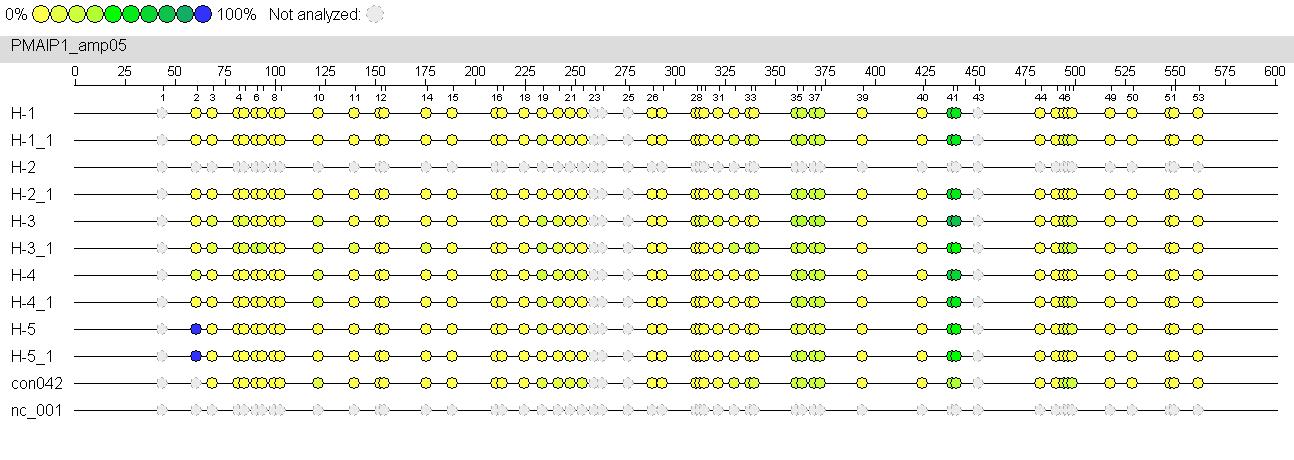
**

**G**

**
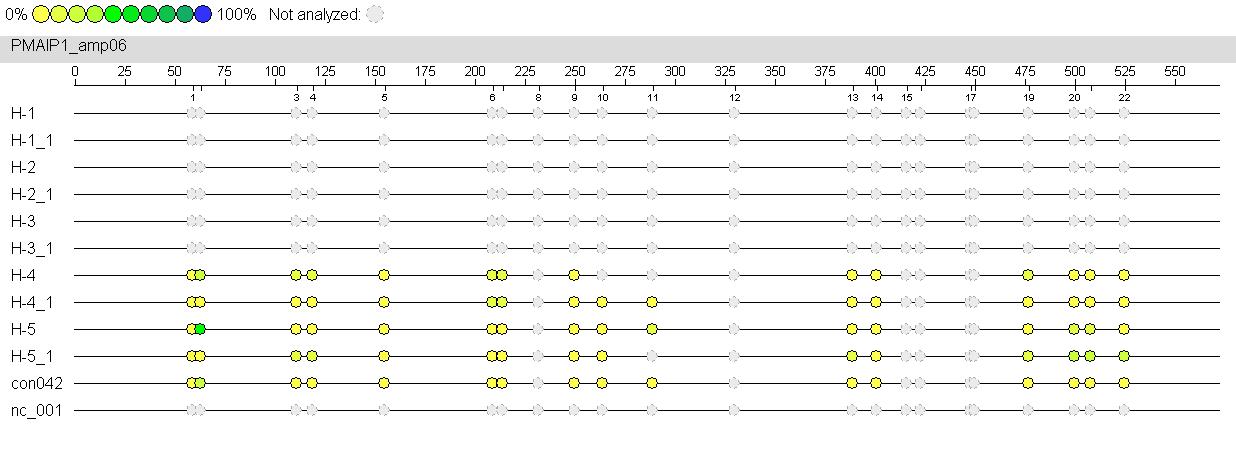
**

**H**

**
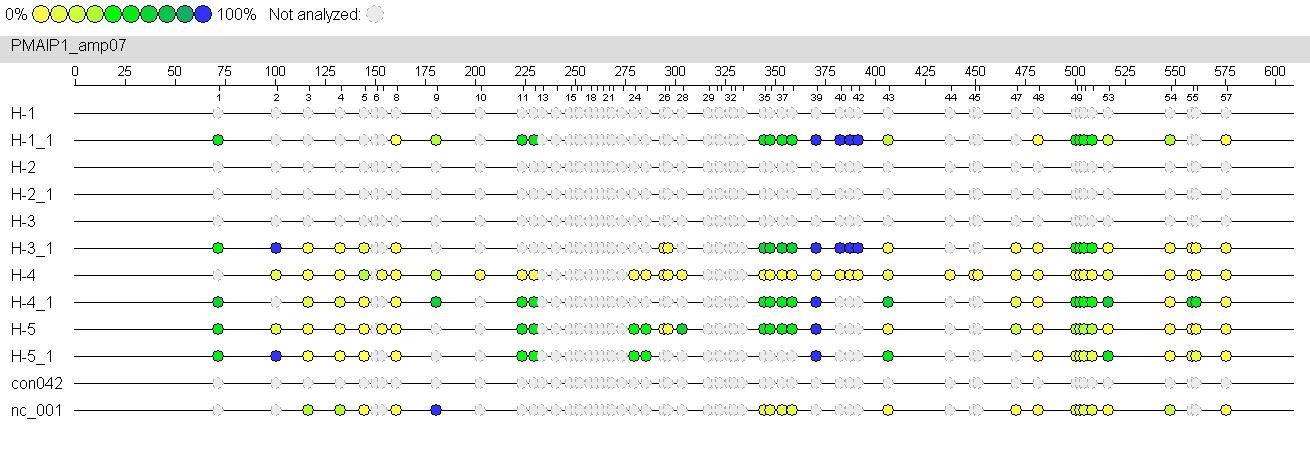
**

**I**

**
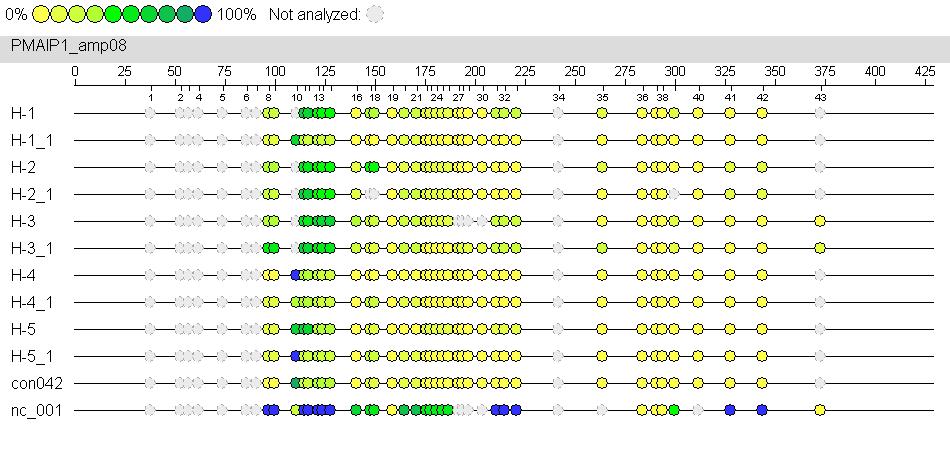
**

Supplement: Additional file 1 — Figure S1. Position of amplicons within the analysed genomic region (A) and methylation status of CpG islands (B-H) of the human PMAIP gene. A, The genomic region of the PMAIP gene is located from −390 to +1153 relative to the gene start. This refers to position 57566802–57568344 of the NCBI’s human genome build 37.1. The gene is shown in its transcripted orientation and locates on the sense strand of chromosome 18. Colors illustrate position of the gene (blue), mRNA (green), region for amplicon design (orange), amplicons (yellow) and annotated (Ensembl) regulatory region (pink). B-H, Colored dots indicate the methylation ratio (%) at each analyzed CpG-unit within each amplicon. Samples are indicated as H-1 to H-5 (time point T0) and H-1_1 to H-5_1 (T14). Base count (upper ruler scale) and CpG-site numbering (lower ruler scale) refers to the analyzed strand in 5’→3’ orientation of the analyzed amplicon sequence. Sample “nc_001” represents the reaction negative control (water) and “con42” a control DNA. [file 1756-0500-5-540-S1.doc]

**Figure S2 (MMD)**

**A**


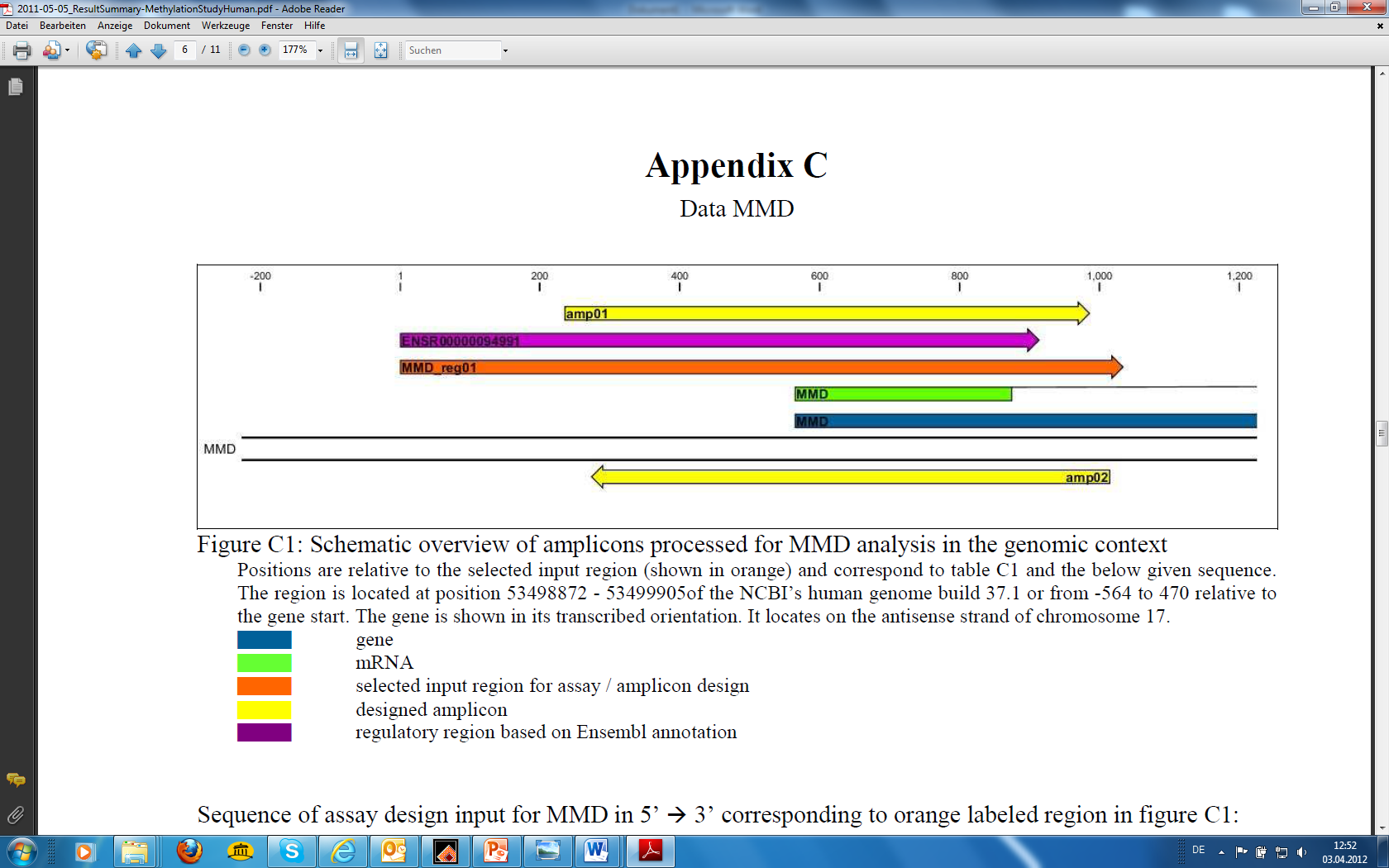


**B**


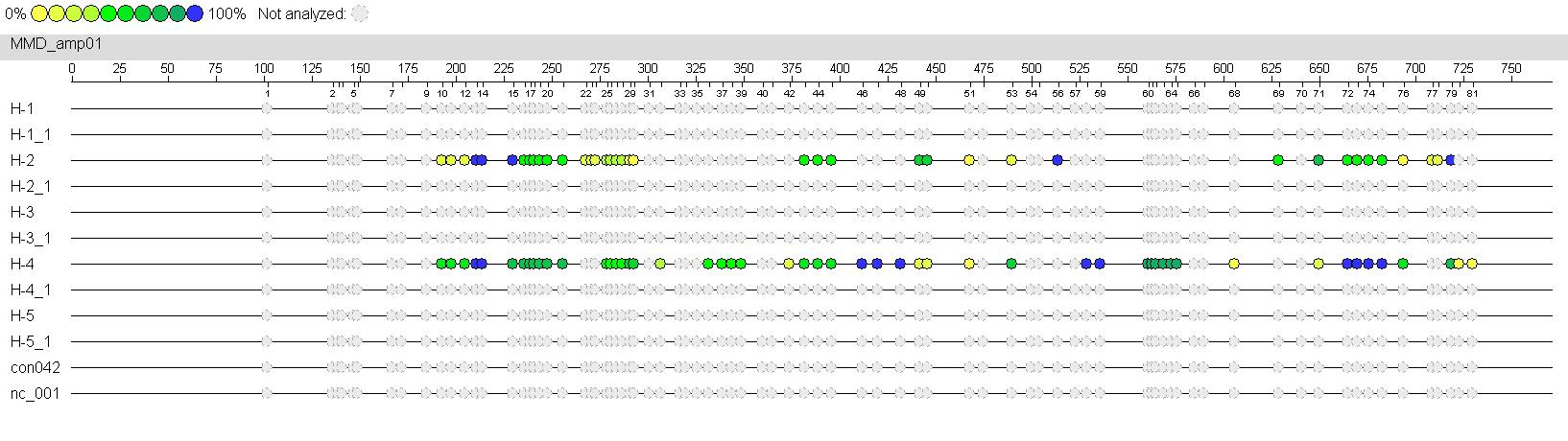


**C**


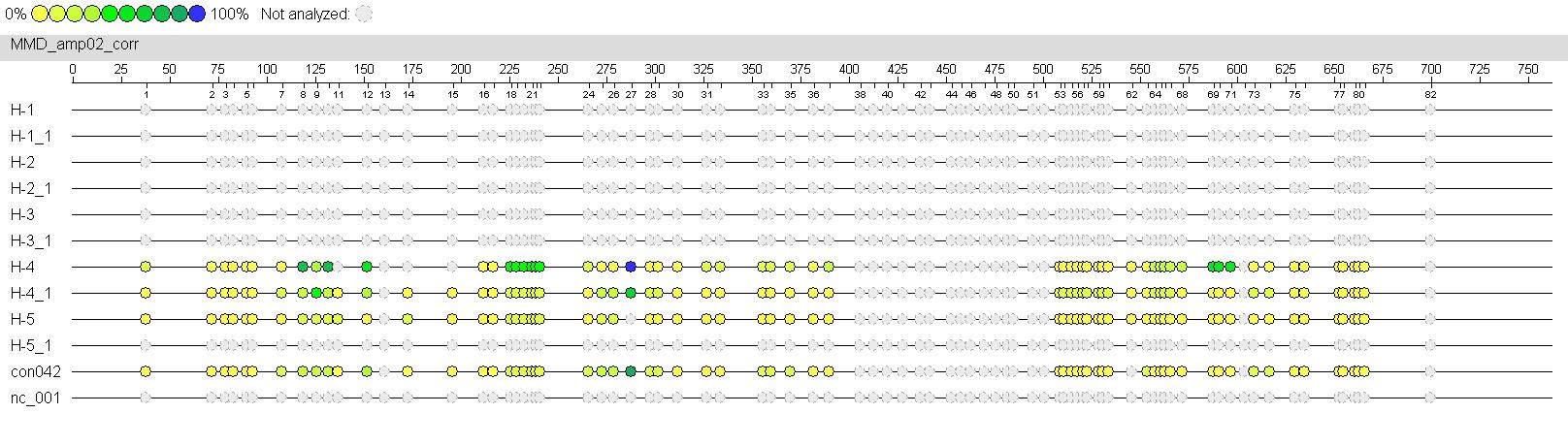

Supplement: Additional file 2 — Figure S2. Position of amplicons with in the analysed genomic region (A) and methylation status of CpG islands (B, C) of the human MMD gene. A, The genomic region of the MMD gene is located from −564 to +470 relative to the gene start. This refers to position 53498872–53499905 of the NCBI’s human genome build 37.1. The gene is shown in its transcripted orientation and locates on the sense strand of chromosome 17. Colors illustrate position of the gene (blue), mRNA (green), region for amplicon design (orange), amplicons (yellow) and annotated (Ensembl) regulatory region (pink). B and C, Colored dots indicate the methylation ratio (%) at each analyzed CpG-unit within each amplicon. Samples are indicated as H-1 to H-5 (time point T0) and H-1_1 to H-5_1 (T14). Base count (upper ruler scale) and CpG-site numbering (lower ruler scale) refers to the analyzed strand in 5’→3’ orientation of the analyzed amplicon sequence. Sample “nc_001” represents the reaction negative control (water) and “con42” a control DNA. [file 1756-0500-5-540-S2.doc]
